# Lack of Association Between Glucose Homeostasis and Immune Checkpoint Inhibitor Outcomes: A Retrospective Institutional Review

**DOI:** 10.3390/cancers17193230

**Published:** 2025-10-04

**Authors:** Joy Justice, Hannah Burnette, Rebecca Irlmeier, Fei Ye, Douglas B. Johnson

**Affiliations:** 1 School of Medicine, Vanderbilt University, 1161 21st Ave. S, Nashville, TN 37232, USA; joy.m.justice@vanderbilt.edu; 2 Department of Medicine, Vanderbilt-Ingram Cancer Center, 2220 Pierce Ave., Nashville, TN 37232, USA; hannah.r.burnette@vumc.org; 3 Department of Biostatistics, Vanderbilt University Medical Center, 1211 Medical Center Dr., Nashville, TN 37203, USA; rebecca.irlmeier@vumc.org (R.I.); fei.ye@vumc.org (F.Y.); 4 Department of Public Health Sciences, Sylvester Comprehensive Cancer Center at the University of Miami, 1475 NW 12th Ave., Miami, FL 33136, USA

**Keywords:** immune checkpoint inhibitors, immunotherapy, CTLA-4, PD-1, metastatic melanoma, patient outcome, treatment toxicity

## Abstract

Background/Objectives: Immune checkpoint inhibitors (ICIs) have revolutionized outcomes for patients with melanoma. As such, it is important to understand factors that may influence response as well as toxicity to these therapies. Impaired glucose control is often a sign of pathologic inflammation and may alter immune system regulation, but it is unclear whether glucose control impacts patients with melanoma on ICIs. Methods: After reviewing patients with melanoma treated with ICIs at our institution between 2014 and 2024, we assessed whether longitudinal glucose control is associated with patient outcomes (response, progression-free survival, overall survival, and treatment toxicity) during ICI therapy. Results: There was no significant difference in baseline glucose values between responders and non-responders (102.5 vs. 106.0, *p* = 0.093). Having a baseline glucose over 200 or any glucose over 200 was not significantly associated with response (*p* = 0.79, *p* = 0.20), progression-free survival (*p* = 0.64, *p* = 0.45), overall survival (*p* = 0.56, *p* = 0.36), or toxicity (*p* = 0.29, *p* = 0.11). Although a diagnosis of diabetes mellitus was not significantly associated with response (*p* = 0.84), progression-free survival (*p* = 0.12), or toxicity (*p* = 0.11), it was associated with improved overall survival (*p* = 0.0034) in the small number of patients with diabetes. Conclusions: Overall, we observed that glucose control was not strongly associated with efficacy or toxicity in patients treated with ICIs.

## Simple Summary

Because immune checkpoint inhibitors (ICIs) drastically improved outcomes for patients with melanoma, it is important to understand factors that may influence patients’ response to therapy. The goal of our retrospective study was to evaluate the relationship between blood glucose levels and outcome in adult patients with melanoma on immunotherapy. In this population, we determined that blood glucose homeostasis does not associate with patient response, disease progression, or the presence of immune-related adverse events. This may influence the monitoring and management of blood glucose values in patients with melanoma on immunotherapy.

## 1. Introduction

Melanoma was predicted to be the fifth most common cancer diagnosed in both men and women in 2024, characterized by a lifetime risk of 1 in 37 men and 1 in 56 women [1,2,3]. Although localized melanoma is generally treated with surgical resection, patients with stage IIB to stage IV melanoma may undergo systemic therapy to address residual or metastatic disease [4]. Prior to the advent of current first-line therapeutic options, it was documented that the median overall survival (OS) for patients with advanced melanoma was less than one year [5,6]. Immune checkpoint inhibitors (ICIs) have significantly improved outcomes for patients with advanced melanoma, and these systemic therapies provide durable responses in a sizable fraction of treated patients.

ICI therapies for melanoma include PD-1 inhibitors (pembrolizumab or nivolumab), CTLA-4 inhibitors (ipilimumab), and LAG-3 inhibitors (relatlimab). These agents facilitate effective immune-mediated elimination of tumor cells, leading to improved survival in patients with melanoma [7,8]. A randomized controlled trial of ICI therapies determined a median OS of 71.9 months in patients treated with a combination of nivolumab + ipilimumab, 36.9 months in patients treated with nivolumab, and 19.9 months in patients treated with ipilimumab; combination therapy was associated with median melanoma-specific survival of more than 120 months [9]. Currently, these agents are often used in the neoadjuvant and adjuvant setting as well, improving event-free survival in this context and preventing the development of metastatic disease. While ICI therapy often produces durable efficacy, these medications also may result in significant immune related adverse events (irAEs), which affect several organ systems and may be severe [10]. Interestingly, patients with melanoma who experience irAEs during ICI therapy experience greater OS, progression-free survival (PFS), and recurrence-free survival (RFS) [11,12,13]. However, approximately half of patients never experience a response to ICI therapy.

Due to the critical role ICIs play in the management of melanoma, it is necessary to understand which factors correlate with their clinical outcomes. Additionally, as patients experience more favorable overall survival (due to durable responses in the metastatic setting and/or increased use in earlier stages of disease), identifying off-target effects, which may have long term consequences, is an important goal. We selected to consider glucose homeostasis due to its multisystem impact on the body. Prior studies have demonstrated that hyperglycemia is associated with activation of proinflammatory pathways, and dysregulated glucose may impair optimal functioning of the immune system [14,15]. Notably, patients with melanoma and concomitant pre-treatment diabetes have a higher neutrophil-to-lymphocyte ratio than those without diabetes, as well as alterations in immune pathway regulation [16]. Hyperglycemia in type II diabetes has also been associated with dysregulation in T-cell functioning [17]. However, the impact of hyperglycemia on outcome in patients on ICI therapy for melanoma is not well understood. Here, we examined whether glucose dysregulation is associated with ICI efficacy and safety by evaluating patient response, overall survival, progression-free survival, and irAEs.

## 2. Materials and Methods

### 2.1. Study Design and Patient Population

**Figure 1:**
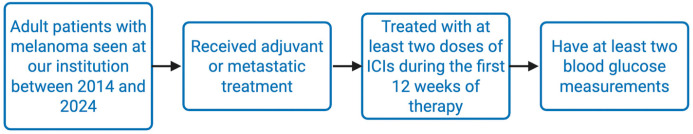
Patient Population Included in our Retrospective Review.

Retrospective review of the electronic medical record was performed of patients with melanoma who were seen either for adjuvant or metastatic treatment at our institution from 2014 to 2024 and who received at least two doses of ICIs during the first 12 weeks of therapy (Figure 1). Patients were excluded who did not have at least two blood glucose measurements during this period. This study received approval from the Vanderbilt University Medical Center Institutional Review Board (#150625).

### 2.2. Study Variables

We collected demographic data, which included patient age at treatment initiation and patient sex from the electronic medical record. ICI treatment, prior therapy for melanoma, tumor stage, LDH level, diagnosis of diabetes mellitus (DM) (if applicable), blood glucose levels, and the use of steroids during the first 12 weeks of therapy were also collected. Difference in glucose (from baseline to the final glucose reading at or prior to 12 weeks of treatment) was assessed and categorized as >10% decrease, 10% decrease to 10% increase, or >10% increase in glucose value. Glucose values were obtained as part of standard laboratory monitoring before and during ICI treatment, and these values were generally non-fasting. All variables were collected by review of the electronic medical record.

ICI treatment was defined as monotherapy if nivolumab or pembrolizumab were used alone, or as combination therapy if nivolumab + ipilimumab were administered concurrently. Information on DM diagnosis was also collected when applicable, including those with a diagnosis of DM at the start of therapy, development of a new diagnosis of DM by 12 weeks of therapy, or not having any diagnosis of DM. We assessed whether patients received any systemic corticosteroids, either for irAEs or other clinical reasons, as a binary variable.

To evaluate patient outcome, we recorded response, progression (yes/no), PFS, OS, patient death (yes/no), and treatment toxicity (yes/no). Response was determined using the RECIST guidelines (version 1.1), which was then partitioned into two groups: responders (complete response and partial response) and non-responders (progressive disease or stable disease). Response was only gauged on patients with metastatic disease whereas PFS, OS, and toxicities were assessed on patients who received both adjuvant therapy and treatment for metastatic disease.

### 2.3. Statistical Analysis

Patient and treatment characteristics were summarized using descriptive statistics. Univariate analyses were conducted to evaluate associations between clinical outcomes (response, PFS, OS, irAEs) and several groupings, including baseline glucose greater than 200 mg/dL, any glucose measurement greater than 200 mg/dL, change in glucose from baseline to final reading, and history of diabetes. Categorical variables were analyzed using Chi-squared tests, while time-to-event outcomes were evaluated using Kaplan–Meier survival curves with log-rank tests.

Multivariable analyses were performed using logistic regression for binary outcomes, response, and irAEs, and Cox proportional hazards regression was used for OS and PFS. Glucose levels were modeled as continuous variables using three approaches in separate models: (1) baseline glucose measurement, (2) change in glucose from baseline to final reading (up to 12 weeks), and (3) median glucose level across all measurements. Additional covariates included monotherapy vs. combination therapy, age, sex, prior therapies, disease stage, LDH level, diabetes status, and additional medications. Complete-case analysis was applied, as missingness in independent variables was minimal. All statistical analyses were conducted using R version 4.4.2.

## 3. Results

### 3.1. Personal and Clinical Demographics

This study included 603 patients with melanoma on ICI therapy, of which 394 (65.3%) received ICI monotherapy and 209 (34.7%) received combination therapy. The median age at initiation of therapy was 63.0 years (Q1 52.0, Q3 73.0), 213 patients (35.3%) were female, and 207 patients (34.3%) had received prior systemic therapy (Table 1). One-hundred twenty-one patients (20.1%) had stage III disease, 197 patients (32.7%) had stage IV M1a-b, and 285 patients (47.3%) had stage IV M1c-d. Thirteen patients (2.2%) had a diagnosis of DM at the start of treatment, and 10 additional patients (1.7%) had a diagnosis of DM by 12 weeks into ICI therapy. Corticosteroids were administered in 272 patients (45.1%).

### 3.2. Blood Glucose Levels and Response Data

Median baseline and 12-week glucose values were 106 and 109, respectively, among all patients, similar between monotherapy (107 and 108) and combination therapy (103.5 and 110). Median baseline and 12-week glucose values were 107 and 107 for patients who required steroids, while median baseline and 12-week glucose values were 104 and 110.5 for patients who did not require steroids. Blood glucose decreased more than 10% in 151 patients (25.0%), increased more than 10% in 169 patients (28.0%), and minimally changed (−10% to +10%) in 283 patients (46.9%).

### 3.3. Univariate Analysis

**Figure 2:**
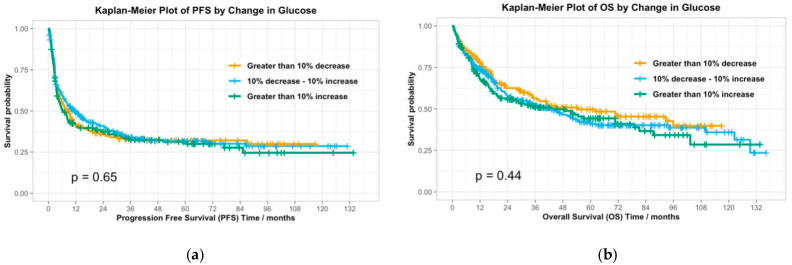
Kaplan–Meier Plots of (**a**) Progression-Free Survival and (**b**) Overall Survival by Change in Glucose.

Among the 522 patients with evaluable response, 218 (41.7%) experienced objective responses. Among all 603 patients, median PFS was 8.2 months (Q1 2.7, Q3 33.2), and median OS was 22.7 months (Q1 8.9, Q3 57.4). IrAEs of any grade occurred in 419 patients (69.5%). There was no significant difference in median baseline blood glucose values between responders and non-responders (102.5 vs. 106.0, *p* = 0.093) (Table 2). Baseline blood glucose over 200 (*n* = 41, 6.8%) or any blood glucose value over 200 (*n* = 135, 22.0%) was not significantly associated with response (*p* = 0.79; *p* = 0.20), PFS (*p* = 0.64; *p* = 0.45), OS (*p* = 0.56; *p* = 0.36), or toxicity (*p* = 0.29; *p* = 0.11). Change in glucose during treatment was also not significantly associated with response (*p* = 0.74), progression-free survival (*p* = 0.65), overall survival (*p* = 0.44) (Figure 2), or toxicity (*p* = 0.67). A diagnosis of DM anytime during treatment (*n* = 23, 3.8%) was not significantly associated with response (*p* = 0.84), PFS (0.12), or toxicity (*p* = 0.11), but these patients had improved overall survival (*p* = 0.0034).

### 3.4. Adjusted Analysis

We performed multivariable analyses to adjust for potential confounding variables. When controlling for patient age, patient sex, monotherapy versus combination therapy, receipt of prior therapies, tumor stage, LDH level, diagnosis of DM, and use of corticosteroids, logistic regression demonstrated no association between response and baseline blood glucose (*p* = 0.30), change in blood glucose (*p* = 0.74), or median blood glucose (*p* = 0.58). Similarly, controlling for these variables, cox regression analysis also did not identify a significant association between PFS or OS and baseline blood glucose (*p* = 0.19, *p* = 0.22), change in blood glucose (*p* = 0.39, *p* = 0.97), or median blood glucose (*p* = 0.75, *p* = 0.39). Lastly, irAEs were not associated with baseline blood glucose (*p* = 0.76), change in blood glucose (*p* = 0.73), or median blood glucose (*p* = 0.92).

## 4. Discussion

To evaluate the association between blood glucose values and outcome in patients with melanoma on ICI therapy, we conducted a retrospective review of all adult patients with melanoma on ICI therapy at VUMC between 2014 and 2024. In this study, we found no obvious or consistent association between blood glucose values (including baseline and changes early in therapy) and response, PFS, OS, or treatment toxicity. Additionally, having a diagnosis of DM was not associated with patient outcome, PFS, and treatment toxicity. However, patients with a diagnosis of DM had a significantly better OS, although the number of patients was quite small. Overall, these findings suggest that strict glucose control is not obviously associated with clinical outcomes in patients with melanoma on immunotherapy.

Prior studies have suggested that patients with a diagnosis of type II DM experience an inferior response to ICI therapy [17,18,19,20]. A recent study by Jan et al. noted a significant difference in OS for patients with concomitant type II DM and melanoma on ICI therapy (median OS 28.5 months with DM vs. 67.3 months without DM, *p* < 0.001), with a similar decrease in survival identified in diabetic patients treated with ICI therapy for non-small cell lung cancer [18,21,22]. Our study was distinct in that it was either larger than most other studies (with one exception [18]) or provided a more granular assessment of baseline and dynamic changes in glucose. This potentially allows us to identify more subtle changes in glucose (e.g., in patients with early insulin resistance). Notably, our study found improvement in OS in patients with type II DM, which could be due to the small number of patients in our study who had been diagnosed with type II DM (*n* = 23). While this association can be utilized to generate further hypotheses, meaningful interpretation is currently limited due to the minimal sample size. As previously mentioned, tumoral transcriptomic profiling has noted differences in innate and adaptive immune pathway regulation in patients with concomitant melanoma and type II DM [16]. Further exploration of the cellular impact of type II DM in patients on ICI therapy would be beneficial.

In the absence of systematic hemoglobin A1c or glucose tolerance testing (which is not routinely performed in oncology clinics as standard of care), it is not entirely clear what method is best for assessing impaired glucose control. Thus, our study considered a variety of variables to represent glucose homeostasis, identifying patients with (1) a prior diagnosis of diabetes, (2) an elevated glucose at baseline, (3) increasing glucose values over time, and (4) an increased glucose at any time, such as with stressors. We largely found negative results, which supports a lack of association between glucose control and clinical outcomes. Our lack of a gold standard measurement for glucose values increases the difficulty of stating definitive conclusions.

Of note, we also did not find an obvious relationship in the other direction; there was no noted relationship between ICI therapy and impaired glucose levels. There was a possible indication that combination ICI therapy was associated with increased glucose values from baseline to 12 weeks (103.5 to 110), and one could hypothesize that this was due to increased use of corticosteroids in this group. However, patients who were not treated with corticosteroids demonstrated more of a trend towards increased glucose than those treated with corticosteroids. Thus, it is conceivable that ICI-induced inflammation resulted in impaired glucose tolerance and early signals for hyperglycemia. Because of our non-significant differences, studies with larger numbers and better controlled variables, ideally with systematically obtained fasting glucose measurements, would be needed to confirm our findings.

More broadly, identifying modest changes in important cardiometabolic parameters could have a large population-wide impact, as ICIs are being used more commonly and in increasingly aggressive combinations. For example, we found that combination ICI therapy was associated with modestly increased systolic blood pressure and atherosclerotic plaque evolution [23,24]. In particular, it would be interesting to evaluate other metabolic parameters (such as having a diagnosis of metabolic syndrome) and their impact on outcomes, although this was beyond the scope of our study; most patients included in our study did not have lipid values or waist circumference assessment. Similarly, several studies have found that increased body mass index correlated with improved outcomes and overall similar toxicity profiles in patients with melanoma treated with ICI therapy, although results have been somewhat discordant [25,26,27]. Studies evaluating lipid levels have also described an association between improved survival and elevated cholesterol values [28,29,30], although other studies have suggested that increased cholesterol in the tumor microenvironment may induce CD8+ T-cell expression of immune checkpoints and decrease the efficacy of immunotherapy [31,32].

Strengths of this study include the large number of patients who were reviewed, the inclusion of patients over a 10-year period, and the granularity of the data involved. Rather than navigating a population-based database, we were able to capture individual glucose values for a large cohort of patients with melanoma on ICI therapy. However, our study is limited by taking place at a single institution and may lack generalizability to other settings or populations. Our tertiary care center may overrepresent patients with more complex diseases, who may have failed prior treatment or have insurance coverage greater than the general population. Due to the demographics of our referral center population, our study may also underrepresent patients of racial or ethnic minorities, as our surrounding region is predominantly white. Because our study is retrospective, we are also unable to assess causality. Lastly, we used glucose values taken in the context of clinical care for cancer, which generally does not require fasting glucose levels. Future evaluations may benefit from obtaining a standardized fasting glucose measurement. In summary, these results suggest that baseline and dynamic glucose changes are not strongly associated with ICI outcomes. Further investigation in multi-center, prospective studies could be considered to confirm these findings.

## 5. Conclusions

Immune checkpoint inhibitors, such as PD-1 inhibitors (pembrolizumab, nivolumab) and CTLA-4 inhibitors (ipilimumab), are the predominant management option for patients with advanced melanoma. By assessing patients’ blood glucose values during the first twelve weeks of immunotherapy, our study analyzed the relationship between glucose homeostasis and efficacy and toxicity outcomes in patients with melanoma on ICIs. We determined that glucose control did not have a significant impact on outcome or survival in this patient population. Prospective evaluation would be beneficial to further explore the relationship between blood glucose homeostasis and outcome in patients with melanoma on immunotherapy. 

## Figures and Tables

**Table 1:** Personal and Clinical Demographic Information.

	Monotherapy (*n*, %) (*n* = 394)	Combination Therapy (*n*, %) (*n* = 209)	Total (*n*, %) (*n* = 603)
**Treatment**			
Nivolumab Pembrolizumab Nivolumab + Ipilimumab	102 (25.6) 292 (74.1) 0 (0)	0 (0) 0 (0) 209 (100)	102 (16.9) 292 (48.4) 209 (34.7)
**Age**			
Mean (SD) * Median [Q1, Q3] Missing Value	63.9 (13.8) 66.0 [55.0, 74.0] 0 (0)	58.0 (14.2) 61.0 [48.0, 68.5] 2 (1.0)	61.9 (14.2) 63.0 [52.0, 73.0] 2 (0.3)
**Sex**			
Female Male	148 (37.6) 246 (62.4)	65 (31.1) 144 (68.9)	213 (35.3) 390 (64.7)
**Prior Therapy**			
Yes No	137 (34.8) 257 (65.2)	70 (33.5) 139 (66.5)	207 (34.3) 396 (65.7)
**Stage**			
Stage III Stage IV M1a/b Stage IV M1c/d	116 (29.4) 123 (31.2) 155 (39.3)	5 (2.4) 74 (35.4) 130 (62.2)	121 (20.1) 197 (32.7) 285 (47.3)
LDH * (U/L)			
≤225 >225 Missing Value	232 (58.9) 142 (36.0) 20 (5.1)	91 (43.5) 112 (53.6) 6 (2.9)	323 (53.6) 254 (42.1) 26 (4.3)
**Diabetes Diagnosis**			
Diabetes at Start Diabetes at 12 Weeks No Diabetes	7 (1.8) 6 (1.5) 381 (96.7)	6 (2.9) 4 (1.9) 199 (95.2)	13 (2.2) 10 (1.7) 580 (96.2)
**Required Steroids**			
Yes No Missing	112 (31.0) 270 (68.5) 2 (0.5)	150 (71.8) 59 (28.2) 0 (0)	272 (45.1) 329 (54.6) 2 (0.3)

* Abbreviations: SD, standard deviation; LDH, lactate dehydrogenase.

**Table 2:** Associations Between Glucose Variables and Outcomes by Univariate Analysis.

	Response (*n*, %)	Median PFS * [Q1, Q3]	Median OS * [Q1, Q3]	Any irAE * (*n*, %)
Baseline Glucose > 200 (*n* = 41)	12 (29.3)	9.4 months [3.3, 42.3]	23.0 months [8.7, 49.7]	32 (78)
Baseline Glucose ≤ 200 (*n* = 499)	180 (36.1)	8.0 months [2.6, 33.6]	23.1 months [8.9, 58.2]	343 (68.7)
*p*-value	0.79	0.64	0.56	0.29
Any Glucose > 200 (*n* = 135)	42 (31.1)	6.1 months [2.3, 21.3]	17.4 months [6.6, 50.7]	102 (75.6)
All Glucose ≤ 200 (*n =* 468)	176 (37.6)	8.3 months [2.7, 35.2]	23.5 months [9.8, 59.2]	317 (67.7)
*p*-value	0.20	0.45	0.36	0.11
>10% Decrease in Glucose (*n* = 151)	54 (35.8)	8.2 months [2.5, 30.7]	28.0 months [10.5, 61.7]	108 (71.5)
10% Decrease to 10% Increase (*n* = 283)	104 (36.7)	9.9 months [2.8, 35.1]	22.7 months [9.5, 58.1]	192 (67.8)
>10% Increase in Glucose (*n* = 169)	60 (35.5)	5.7 months [2.6, 29.1]	19.3 months [8.1, 52.3]	119 (70.4)
*p*-value	0.74	0.65	0.44	0.67
Diagnosis of Diabetes (*n* = 23)	8 (34.8)	14.9 months [3.7, 38.7]	27.8 months [16.8, 51.5]	20 (87.0)
No Diagnosis of Diabetes (*n* = 580)	210 (36.2)	7.8 months [2.7, 32.6]	21.6 months [8.7, 57.9]	399 (68.8)
*p*-value	0.84	0.12	0.0034	0.11

* Abbreviations: PFS, progression-free survival; OS, overall survival; irAE, immune-related adverse event.

## Data Availability

The data that support the findings of this study are available from the corresponding author, D.J., upon reasonable request.

## Abbreviations

The following abbreviations are used in this manuscript:

ICIs	Immune checkpoint inhibitors
OS	Overall survival
irAEs	Immune-related adverse events
PFS	Progression-free survival
RFS	Recurrence-free survival
DM	Diabetes Mellitus
SD	Standard deviation
LDH	Lactate dehydrogenase
